# Biological Activity of Peptide Fraction Derived from *Hermetia illucens* L. (Diptera: Stratiomyidae) Larvae Haemolymph on Gastric Cancer Cells

**DOI:** 10.3390/ijms26051885

**Published:** 2025-02-22

**Authors:** Roberta Rinaldi, Simona Laurino, Rosanna Salvia, Sabino Russi, Federica De Stefano, Rocco Galasso, Alessandro Sgambato, Carmen Scieuzo, Geppino Falco, Patrizia Falabella

**Affiliations:** 1 Department of Basic and Applied Sciences, University of Basilicata, Via dell’Ateneo Lucano 10, 85100 Potenza, Italy; roberta.rinaldi@unibas.it (R.R.); r.salvia@unibas.it (R.S.); federica.destefano@unibas.it (F.D.S.); 2 Centro di Riferimento Oncologico della Basilicata IRCCS (IRCCS-CROB), 85028 Rionero in Vulture, Italy; simona.laurino@crob.it (S.L.); sabino.russi@crob.it (S.R.); rocco.galasso@crob.it (R.G.); 3 Spinoff XFlies S.R.L, University of Basilicata, Via Dell’Ateneo Lucano 10, 85100 Potenza, Italy; 4 Department of Translational Medicine and Surgery, Università Cattolica del Sacro Cuore, 00168 Rome, Italy; alessandro.sgambato@unicatt.it; 5 Multiplex Spatial Profiling Facility, Fondazione Policlinico Universitario ‘Agostino Gemelli’ IRCCS, 00136 Rome, Italy; 6 Department of Biology, University of Naples Federico II, 80126 Naples, Italy; geppino.falco@unina.it

**Keywords:** black soldier fly, anticancer peptides, haemolymph extracts, in vitro anticancer activity

## Abstract

Gastric cancer (GC) is one of the leading causes of cancer-related mortality worldwide, characterised by poor prognosis and limited responsiveness to chemotherapy. There is a need for new and more effective anticancer agents. Antimicrobial peptides (AMPs) represent a promising class of biomolecules for this purpose. Naturally occurring in the innate immune system, these peptides can also exert cytotoxic effects against cancer cells, earning them the designation of “anticancer peptides” (ACPs). They have the potential to be a viable support for current chemotherapy schedules due to their selectivity against cancer cells and minor propensity to induce chemoresistance in cells. Insects are an excellent source of AMPs. Among them, due to its ability to thrive in hostile and microorganism-rich environments, we isolated a peptide fraction from *Hermetia illucens* L. (Diptera: Stratiomyidae) haemolymph to evaluate a possible anticancer activity. We tested Peptide Fractions (PFs) against AGS and KATO III gastric cancer cell lines. Data obtained indicated that PFs, especially those resulting from *Escherichia coli* and *Micrococcus flavus* infection (to boost immune response), were able to inhibit tumour cell growth by inducing apoptosis or cell cycle arrest in a cell line-specific manner. These results support further investigation into the use of antimicrobial peptides produced from insects as possible anticancer agents.

## 1. Introduction

Cancer is among the primary causes of mortality globally, typified by uncontrolled proliferation of cells and multiple mutations [1,2,3,4,5,6,7]. In particular, gastric cancer (GC) is a multifactorial disease whose onset and progression may be influenced by both hereditary and environmental factors. The median age at diagnosis is 70 years, with incidence rates increasing progressively with age; however, approximately 10% of stomach cancer cases occur in patients under 45 [8]. Since young patients are less exposed to environmental carcinogens, early-onset gastric cancer is a valuable model to investigate genetic changes associated with the multi-stage process of carcinogenesis, characterised by the progressive development of mutations and epigenetic alterations in the expression of various genes responsible for the development of the disease [9,10].

Tumour progression, a prolonged process unfolding over decades, involves the gradual acquisition of neoplastic characteristics by normal cells [11]. This transformation is driven by stochastic mutations and epigenetic modifications that disrupt regulatory pathways governing proliferation, survival, apoptosis, cellular differentiation, and other hallmarks of malignancy, enabling malignant cells to bypass normal growth constraints [12]. These genetic and epigenetic alterations not only confer a survival advantage but also facilitate the emergence of other hallmark traits, such as angiogenesis and immune evasion. The complexity of this process reflects the breakdown of tightly regulated genomic and epigenomic mechanisms essential for maintaining cellular homeostasis and tissue integrity [13].

Various therapeutic approaches exist for cancer treatment, and for GC in particular, but many of these are ineffective due to the multidrug resistance (MDR) phenomenon, which remains a major challenge in effective therapeutic interventions [14,15]. In oncology, MDR is defined as the ability of cancer cells to survive treatment with a range of anticancer drugs, similar to the concept commonly applied to antibiotic treatment [16]. A growing number of studies indicate that MDR is mediated by alterations in the tumour microenvironment (TME), resulting in increased efflux of chemotherapeutic drugs (which decreases drug uptake by tumour cells), changes in drug metabolism and drug targets, reduced drug efflux, suppression of apoptosis, enhanced DNA repair mechanisms, oncogenic mutations, and tumour heterogeneity [17,18].

Several methods have been proposed in recent decades to overcome resistance and increase the effectiveness of cancer treatments. General approaches to drug resistance include early tumour diagnosis, active monitoring during therapy, adding new drugs and improving pharmacologic principles, integrating clinical-genomic data, identifying new antibodies and biomarkers, and immunotherapy through TME modulation [19,20,21,22,23,24].

In particular, systemic chemotherapy, radiotherapy, surgery, immunotherapy, and targeted therapy have all demonstrated their efficacy in GC; therefore, multidisciplinary treatment is crucial. Furthermore, the classification of gastric cancer according to molecular subtypes offers the opportunity for individualised therapy [25,26].

Antimicrobial peptides (AMPs), small bioactive proteins that all living organisms produce as an essential part of their innate immune system, are of particular interest in this context [27]. AMPs are characterised by a short amino acid sequence (between 10 and 50 amino acids approximately) and a rich variety of primary characteristic structures (e.g., proline-rich, glycine-rich or cysteine-rich AMPs) and secondary structures (such as α-helical AMPs or β-sheet AMPs).

They can have a net charge ranging from −8 (anionic antimicrobial peptides—AAPs) to +10 (cationic antimicrobial peptides—CAPs, rich in lysine and arginine residues) and very strong thermal stability [28]. AMPs have an amphipathic structure, containing both hydrophobic and hydrophilic regions, which enables them to be soluble in aqueous environments [29]. AAPs have a net negative charge of −1 to −8. AAPs include many negatively charged aspartic and glutamic acid residues and are found in many important organs, such as the brain, epidermis, respiratory tract, and gastrointestinal tract. They are the least common class of AMPs [30].

CAPs, on the other hand, are the most common AMPs. They have been shown to act as anticancer agents predominantly on tumour cells and less so on normal healthy cells [31], by destroying tumour cell membranes (membranolytic actions) [32,33,34] or by entering cells and interacting with intracellular components or pathways (non-membranolytic actions) [35]. The secondary structure of AMPs can be modified by increasing the number of positively charged amino acids or changing their position in the peptide chain, and this can increase their antibacterial and antitumour activity [36]. In order to explain the antimicrobial activity of AMPs, membrane interactions have been studied, which are mediated by electrostatic forces between positively charged AMPs and negatively charged microbial surfaces: the teichoic acids of the cell wall of Gram-positive bacteria and the lipopolysaccharides (LPS) of the outer membrane of Gram-negative bacteria charge the electronegatively charged microbial surfaces, enhancing the interaction with AMPs [37].

Tumour cells also have a negative net charge as they express anionic molecules such as phosphatidylserine and O-glycosylated mucins on the outer surface of the cytoplasmic membrane [38], and these same molecules are either not or less expressed on the outer surface of healthy mammalian cell membranes, characterised by a neutral charge at physiological pH, due to the zwitterionic phosphatidylcholine and sphingomyelin predominancy [39,40,41,42]. The negative net charge of the cancer cell membrane allows electrostatic interactions with CAPs. Cancer cells also have greater membrane fluidity and more microvilli on the surface than healthy cells and therefore can interact more effectively with CAPs [43,44,45,46,47]. The electrostatic interaction between cell membrane and AMPs can lead to the membrane disruption with cytoplasmic leakage (membranolytic mechanism) or can allow for the transient pore formation with AMPs entry into the cell, targeting intracellular component (non-membranolytic mechanism) [48]. The intracellular mechanisms of AMPs with anticancer activity include regulation of apoptosis by targeting molecules and pathways involved in carcinogenesis [49]; activation of the mitochondria-dependent pathway generated by signals such as DNA damage, oxidative stress, and lack of growth factors, which simultaneously release pro-apoptotic proteins such as cytochrome C (Cyt C) from the mitochondria to the cytosol [50]; modulation of the proteasome, such as proteasome inhibition, which causes blockage of cell cycle progression in several cancer cell lines [51]; the activation of immune pathways such as natural killer (NK) cells and the interferon (IFN) signalling pathway, as interferons have anti-tumour activities by directly influencing tumour cell proliferation and indirectly affecting immunomodulatory and anti-angiogenic responses, while natural killer cells play an important role in the elimination of malignant cells by decreasing the expression of MHC class I molecules, which are present on a variety of tumour cells [52]. Another important anti-tumour activity of peptides with anticancer activity (known as anticancer peptides—ACPs) may involve cell cycle arrest through interactions with key regulatory proteins such as p53, a transcription factor essential for maintaining genome integrity, whose mutations are frequently associated with uncontrolled cell proliferation and tumour progression [53]. Certain ACPs, similar to the azurin-derived peptide fragment studied by Yamada et al. (2009), may form complexes with p53, stabilizing its active conformation and preventing its inhibitory degradation. This interaction leads to intracellular p53 accumulation, triggering cell cycle arrest and ultimately inducing mitochondrial apoptosis via both caspase-dependent and caspase-independent pathways [54]. AMPs are particularly abundant in several insect species, providing new opportunities for the development of innovative, but yet under-explored, therapies [55]. In particular, the bioconverter *Hermetia illucens* L. (Diptera: Stratiomyidae), also known as Black Soldier Fly (BSF) [56,57,58,59,60,61], thrives in contaminated environments despite lacking an adaptive immune system. This resilience is due to its highly effective innate immune response, which enables the production of a high and diversified number of AMPs [62]. *H. illucens* putative AMPs sequences identified in various transcriptomes were subjected to in silico analysis to assess their chemo-physical characteristics and potential biological activities, and some of them were predicted to have anticancer activity [56].

Since AMPs are preferentially released in the haemolymph in response to microbial infections, in this study, the putative anticancer activity, on gastric cancer cell lines, of peptide fraction (PF) extracted from haemolymph collected from *H. illucens* larvae [57] was investigated. The results obtained suggest the presence of biologically active peptides in the haemolymph. This supports the hypothesis that insect-derived AMPs can have anticancer activity and are worthy of investigation, in order to identify new effective anticancer molecules.

## 2. Results

### 2.1. Reduction of Viability of KATO III and AGS Cell Lines by Peptide Fractions

**Figure 1:**
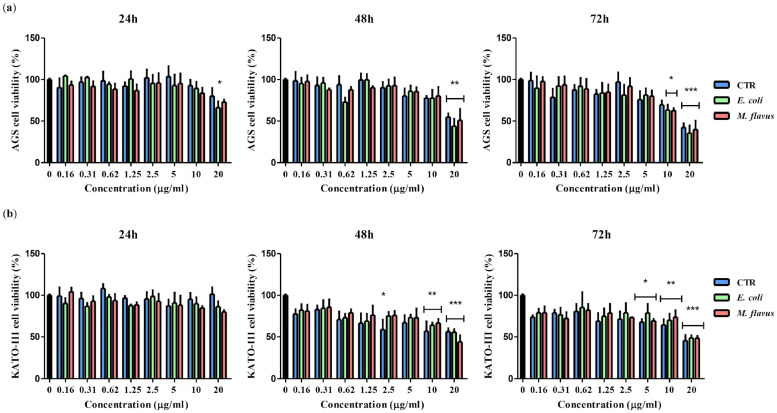
Evaluation of AGS and KATO III cell viability following exposure to PFs at varying concentrations over time. MTT assay was performed after incubation of (**a**) AGS and (**b**) KATO III cells with 20 µg/mL, 10 µg/mL, 5 µg/mL, 2.5 µg/mL, 1.25 µg/mL, 0.62 µg/mL, 0.31 µg/mL, 0.16 µg/mL PFs (PF CTR in blue, PF *E. coli* in green, and PF *M. flavus* in red) for 24 h, 48 h, and 72 h. Cell viability was expressed as a percentage relative to control of untreated cells (black bar). The graphs represent means ± Standard Error (SE) of three independent experiments; differences between treatment groups were estimated by one-way ANOVA followed by Dunnett’s *post hoc* test (* *p* < 0.05, ** *p* < 0.01, *** *p* < 0.001) using GraphPad Prism software 5.0. Significant differences were evaluated vs. untreated cells.

The activity of *H. illucens* PFs derived from larvae infected with *E. coli* (PF *E. coli*), *M. flavus* (PF *M. flavus*), or uninfected larvae (PF CTR), was evaluated through an MTT assay. In particular, KATO III and AGS cells were treated for 24 h, 48 h, and 72 h with doubling concentrations of PFs (0.16 to 20 µg/mL). AGS cell viability was significantly reduced (*p* < 0.01) at 48 h and 20 µg/mL (PF CTR 54.7%; PF *E. coli* 43.9% and PF *M. flavus* 50.9%). The same trend was observed at 72 h (*p* < 0.001) with a viability of 42.3%, 35.6%, and 39.8% for PF CTR, *E. coli* and *M. flavus*, respectively (Figure 1a). On KATO III cells, the activity was similar with a significant inhibition of proliferation (*p* < 0.001) at 10 µg/mL (PF CTR 56.8%; PF *E. coli* 63.9%; PF *M. flavus* 66.5%) and 20 µg/mL (PF CTR 56.3%; PF *E. coli* 55.5%; PF *M. flavus* 44.0%) after 48 h and at 10 µg/mL (PF CTR 64.2%; PF *E. coli* 69.9%; PF *M. flavus* 73.6%) and 20 µg/mL after 72 h of treatment (PF CTR 45.5%; PF *E. coli* 48.6%; PF *M. flavus* 48.4%) (Figure 1b). Overall, PFs exhibited a dose- and time-dependent effect on cell viability in both cell lines.

### 2.2. Cell Morphology Alteration of AGS and KATO III Cell Lines After Exposure to Peptide Fractions

**Figure 2:**
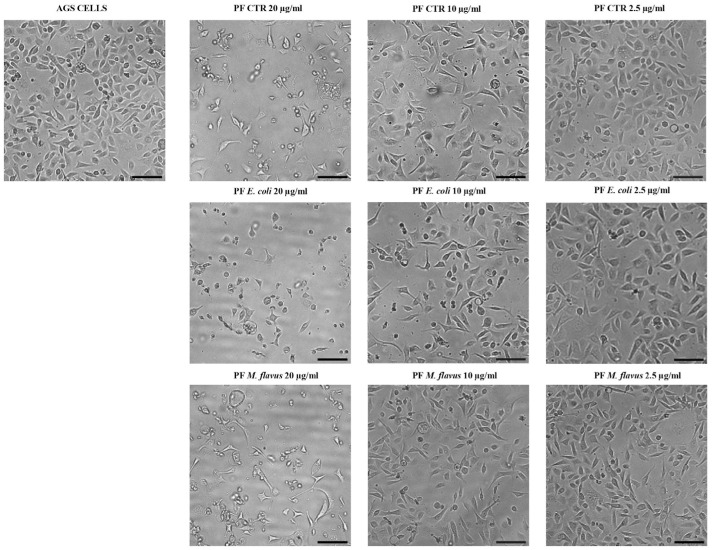
Microscopic analysis of AGS cells treated with PFs. Morphological cell analysis of AGS cell line treated for 48 h with 20 µg/mL, 10 µg/mL, 2.5 µg/mL PFs and untreated, observed through inverted phase contrast microscope. Images represent observations made on each sample in at least three separate fields of view. Magnification: 10×; scale bars: 80 µm.

**Figure 3:**
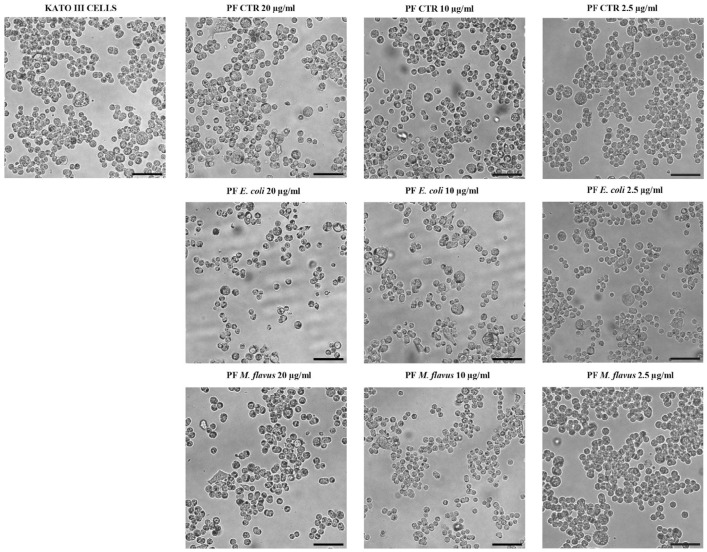
Microscopic analysis of KATO III cells treated with PFs. Morphological cell analysis of KATO III cell line treated for 48 h with 20 µg/mL, 10 µg/mL, 2.5 µg/mL PFs and untreated, observed through inverted phase contrast microscope. Images represent observations made on each sample in at least three separate fields of view. Magnification: 10×; scale bars: 80 µm.

Morphological changes were observed when cells were exposed to 20 µg/mL of PF CTR, PF *E. coli*, and PF *M. flavus*. These changes, compared to untreated cells, included reduced cell size, formation of vacuoles, increased cell detachment, and rounded shape of AGS cells. At concentrations of 10 µg/mL and 2.5 µg/mL, AGS cells were comparable to the untreated control (Figure 2). No significant morphological alterations were observed in KATO III cells (Figure 3).

### 2.3. Apoptosis via Bcl-2/Caspase-3/PARP-1 Pathway in AGS Cell Line Induced by PFs

**Figure 4:**
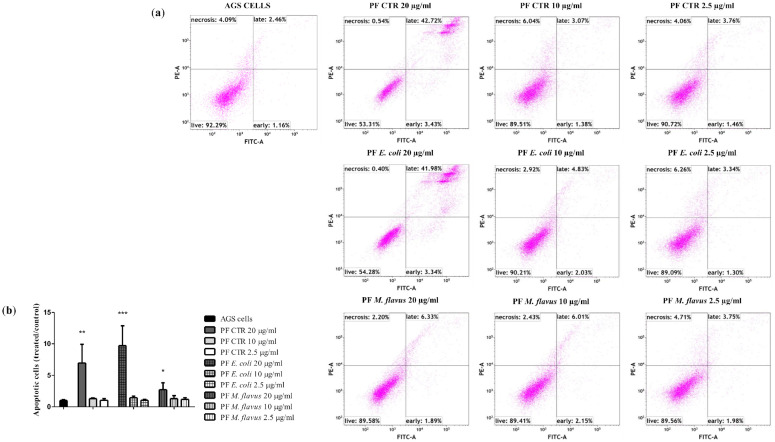
FACS analysis for apoptosis in AGS cell line treated for 48 h with 20 µg/mL, 10 µg/mL, 2.5 µg/mL PFs, and untreated controls. (**a**) The FACS profiles represent 10,000 events and are indicative of the patterns seen in three replications, and (**b**) the resultant histogram analysis shows data as means ± Standard Error (SE) of three independent experiments. Statistical significance was evaluated using GraphPad Prism software 5.0 with one-way ANOVA, followed by Dunnett’s *post hoc* test (* *p* < 0.05, ** *p* < 0.01, *** *p* < 0.001). Significant differences were evaluated vs. untreated cells (AGS cells bar).

**Figure 5:**
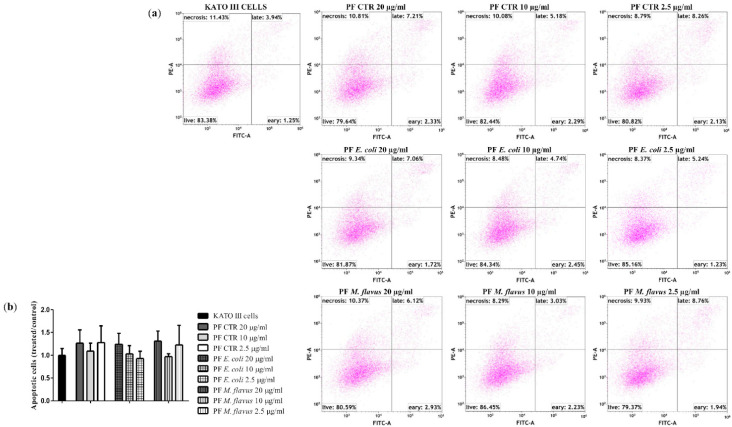
FACS analysis for apoptosis on KATO III cell line treated for 48 h with 20 µg/mL, 10 µg/mL, 2.5 µg/mL PFs, and untreated controls. (**a**) The FACS profiles represent 10,000 events and are indicative of the patterns seen in three replications, and (**b**) the resultant histogram analysis shows data as means ± Standard Error (SE) of three independent experiments. Statistical significance was evaluated using GraphPad Prism software 5.0 with one-way ANOVA, followed by Dunnett’s *post hoc* test. Significant differences were evaluated vs. untreated cells (KATO III cells bar).

To evaluate the mechanisms of cell death, a flow cytometry apoptosis assay was performed on AGS and KATO III cell lines treated with 20 µg/mL, 10 µg/mL, and 2.5 µg/mL of PFs for 48 h. Figure 4 shows an increasing number of apoptotic cells in a dose-dependent manner, in particular for cells treated with 20 µg/mL of PF *E. coli* (18.48%, ** *p* < 0.01) and of PF CTR (20.47%, *** *p* < 0.001), and to a lesser extent for cells treated with 20 µg/mL of PF *M. flavus* (6.41%, * *p* < 0.05), compared with the untreated cells (5.93%). This effect was not observed in KATO III cells under the same conditions (Figure 5).

**Figure 6:**
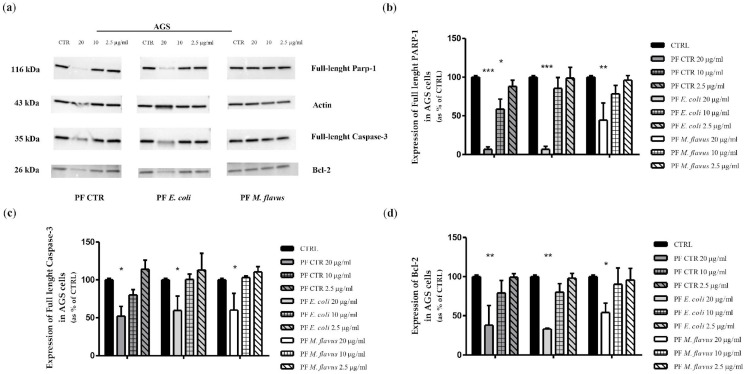
Western blot analysis of apoptosis-related proteins on AGS cells. (**a**) Western blot analysis showing the expression levels of proteins related to the apoptotic pathway, (**b**) PARP1, (**c**) Caspase-3, and (**d**) Bcl-2 in AGS cell line after exposing cells for 48 h with 20, 10, and 2.5 µg/mL of PFs. The relative expression levels were normalised to the housekeeping control, actin. The data are expressed as means ± Standard Error (SE) of three independent experiments. Statistical significance was evaluated using GraphPad Prism software 5.0 with one-way ANOVA, followed by Dunnett’s *post hoc* test (* *p* < 0.05, ** *p* < 0.01, *** *p* < 0.001). Significant differences were evaluated vs. untreated cells. Significant differences were evaluated vs. untreated cells (CRTL).

Apoptosis induction was further assessed via Western blot by evaluating the expression levels of full-length (116 kDa) nuclear poly (ADP-ribose) polymerase (PARP-1), full length (35 kDa) cysteine-aspartic acid protease 3 (Caspase-3), and the anti-apoptotic protein Bcl-2. In AGS cells (Figure 6), both full-length-PARP-1 and full-length Caspase-3 (35 kDa) decreased in a dose-dependent manner.

Additionally, Bcl-2, known as a cell survival protein that inhibits apoptosis, significantly decreased in a dose-dependent manner.

**Figure 7:**
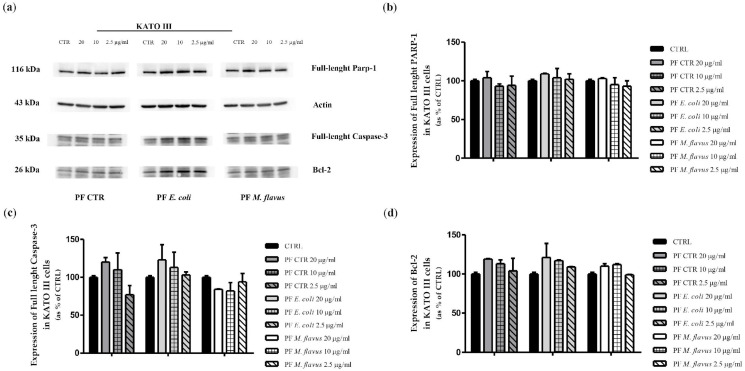
Western blot analysis of apoptosis-related proteins on KATO III cells. (**a**) Western blot analysis showing the expression levels of proteins related to the apoptotic pathway, (**b**) PARP1, (**c**) Caspase-3, and (**d**) Bcl-2 on KATO III cell line after exposing cells for 48 h with 20, 10, and 2.5 µg/mL of PFs. The relative expression levels were normalised to the housekeeping control, actin. The data are expressed as means ± Standard Error (SE) of three independent experiments. Statistical significance was evaluated using GraphPad Prism software 5.0 with one-way ANOVA, followed by Dunnett’s *post hoc* test.. Significant differences were evaluated vs. untreated cells (CTRL).

On the contrary, in KATO III cells (Figure 7), there was no significant change in PARP-1, Caspase-3, and Bcl-2 expressions, in line with results from the cytofluorimetric analysis.

### 2.4. S Phase and G2/M Phase Arrest Induced by Peptide Fractions

**Figure 8:**
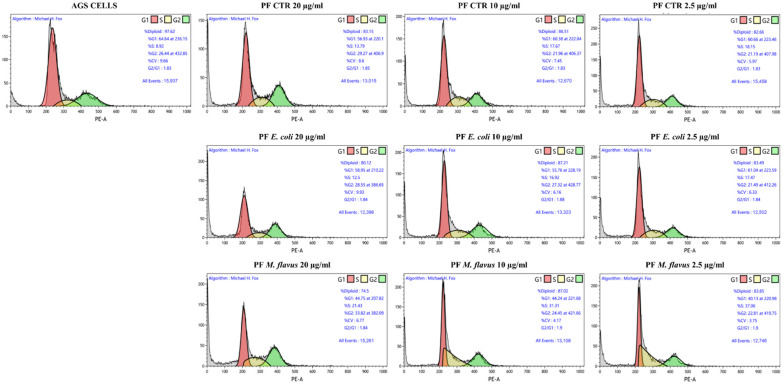
Representative plots (10,000 events) of cell cycle analysis by flow cytometry on AGS cell line. Dot plots report the distribution of cell cycle phases of AGS cells treated with PFs for 48 h at concentrations of 20, 10, and 2.5 µg/mL and untreated.

**Figure 9:**
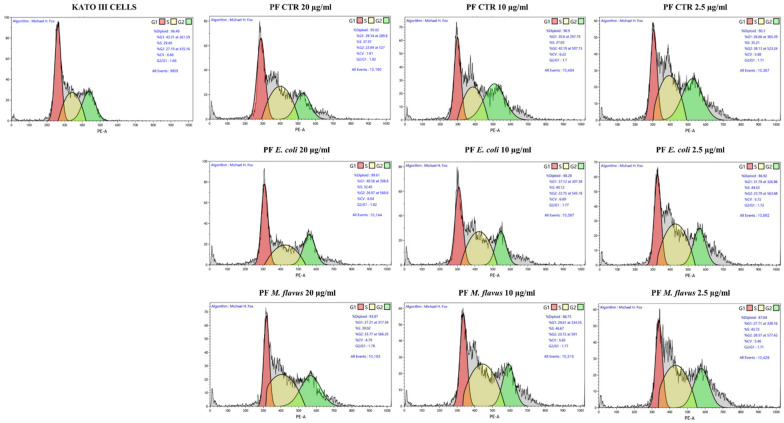
Representative plots (10,000 events) of cell cycle analysis by flow cytometry on KATO III cell line. Dot plots report the distribution of cell cycle phases of KATO III cells treated with PFs for 48 h at concentrations of 20, 10, and 2.5 µg/mL and untreated.

To verify whether the viability reduction after PF treatment was due to cell cycle arrest rather than apoptosis in KATO III, or in addition to apoptosis in AGS, flow cytometry evaluations were performed on both cell lines (Figure 8 and Figure 9).

**Figure 10:**
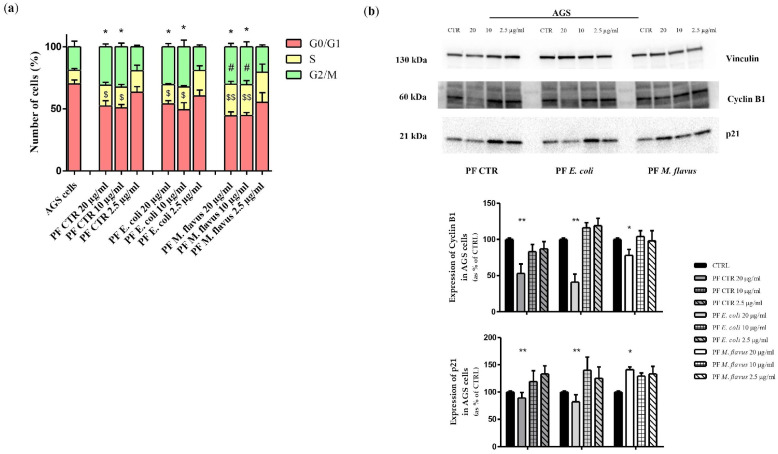
Cell cycle analysis in AGS cells. (**a**) Histogram reports the mean ± Standard Error (SE) of percentage of AGS cells treated with PFs for 48 h at concentrations of 20, 10, and 2.5 µg/mL and untreated in each phase of the cell cycle (G0/G1, S, and G2/M) from three separate experiments. (**b**) Western blot analysis reporting the expression levels of protein related to cell cycle mechanisms (Cyclin B1 and p21) on AGS cells treated with PFs for 48 h at concentrations of 20, 10, and 2.5 µg/mL and untreated. The relative expression levels are normalised to the housekeeping control, vinculin. The data are expressed as means ± Standard Error (SE) of three independent experiments. Statistical significance was evaluated using GraphPad Prism software 5.0 with one-way ANOVA, followed by Dunnett’s *post hoc* test (* *p* < 0.05, ** *p* < 0.01; ^#^ *p* < 0.05 or ^$^ *p* < 0.05, ^$$^ *p* < 0.01). Significant differences were evaluated vs. untreated cells (CTRL).

As shown in Figure 10a, the cell cycle analysis highlighted an accumulation of AGS in G2/M phase (* *p* < 0.05 significance), with a consequent decrease of G0/G1 phase (^$^ *p* < 0.05, ^$$^ *p* < 0.01 significance) after 48 h of treatment (20 and 10 ng/µL of PF CTR and PF *E. coli*), while PF *M. flavus* also determined a significant increase of cells in S phase (^#^ *p* < 0.05 significance).

Western blot analysis of AGS cells lysate (Figure 10b) reported a dose-dependent decrease in cyclin B1, a regulatory protein involved in mitosis and mainly expressed during the G2/M phase of the cell cycle, with the highest concentration of both PF CTR and PF *E. coli*, confirming a G2/M arrest in AGS cell line. The same trend, under the same conditions, was observed for the p21 protein. PF *M. flavus* treatment induced a minor reduction of cyclin B1 levels, but higher levels of p21 than other PFs were observed.

**Figure 11:**
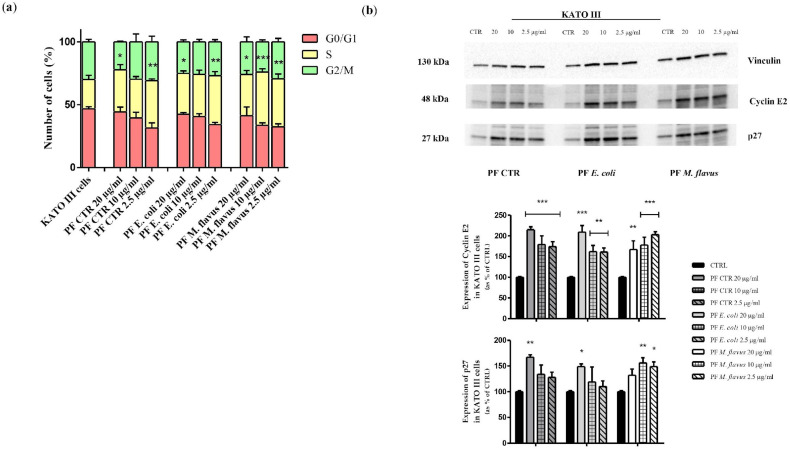
Cell cycle analysis in KATO III cells. (**a**) Histogram reports the mean ± Standard Error (SE) of percentage of treated KATO III cells treated with PFs for 48 h at concentrations of 20, 10, and 2.5 µg/mL and untreated in each phase of the cell cycle (G0/G1, S, and G2/M) from three separate experiments. (**b**) Western blot analysis reporting the expression levels of protein related to cell cycle mechanisms (Cyclin E2 and p27) on KATO III cells treated with PFs for 48 h at concentrations of 20, 10, and 2.5 µg/mL and untreated. The relative expression levels are normalised to the housekeeping control, vinculin. The data are expressed as means ± Standard Error (SE) of three independent experiments. Statistical significance was evaluated using GraphPad Prism software 5.0 with one-way ANOVA, followed by Dunnett’s *post hoc* test (* *p* < 0.05, ** *p* < 0.01, *** *p* < 0.001). Significant differences were evaluated vs. untreated cells (CTRL).

As for KATO III cells (Figure 11a), an accumulation of cells in the S phase was observed after treatment for 48 h with all three types of PFs (* *p* < 0.05, ** *p* < 0.01, *** *p* < 0.001 significance). Figure 11b shows a Western blot analysis of KATO III cells, in which densitometric analysis revealed that there was an increase in cyclin E in cells treated with all PFs. In particular, for cells treated with PF CTR and PF *E. coli* samples, a dose-dependent increase was observed, while those treated with *M. flavus* did not show this effect. In addition, a similar trend was observed for the accumulation of p27 protein levels.

## 3. Discussion

Cancer is one of the leading causes of mortality worldwide, and an increasing number of patients experience treatment resistance or side effects of therapies [16,63,64,65,66].

In particular, in GC (gastric cancer) treatment, chemotherapy often involves a combination of different drugs (e.g., epirubicin, cisplatin and fluorouracil), rather than a single agent [67]. However, surgical removal of the tumour mass is usually the first and most important intervention, depending on the severity of the disease and the degree of stomach involvement [68]. Unfortunately, as is the case with most cancers, the use of chemotherapeutic drugs in stomach cancer treatment has proved to be highly controversial. It seems, indeed, that the administration of some drugs is mainly useful for neoadjuvant purposes, i.e., prior to surgery, and that they may be useful in reducing the tumour extension, increasing the chances of successful treatment. Several treatment options have been developed for clinical use, including radiotherapy, chemotherapy, cancer vaccines, and targeted therapies, which bode well for the treatment of GC, offering new avenues for clinical management and expanding understanding of this type of cancer [15]. Despite these advances, significant challenges remain in therapy development. One major obstacle to targeted therapy is drug resistance in patients, which significantly reduces treatment efficacy. For this reason, targeted therapy and the use of new active molecules are currently among the most promising areas of research for the treatment of GC [69,70].

AMPs are among the most intriguing natural molecules with potential applications in cancer treatment. Although AMPs are produced by all living organisms, insects, accounting for approximately 55% of the total biodiversity on Earth, are among the richest and most innovative sources [46]. Due to this, insects have gained increasing attention in recent years as a source of bioactive molecules with different and putative activities in the biomedical field [48,71,72,73,74,75,76,77,78,79,80,81,82,83,84].

A review by Staczek et al. (2023) [85] underlines that some insect AMPs exhibit selective in vitro cytotoxicity against different cancer cell lines, e.g., melanoma, lymphoma, leukemia, breast cancer, lung cancer, and bladder cancer [86,87,88]. Additionally, many publications have shown the potential in vivo involvement of insect AMPs in antitumour action mostly based on research conducted using a genetically modified *Drosophila melanogaster* model, in which tumour can be induced [89]: tumour cells in *D. melanogaster* activate a cellular response, releasing Tumour Necrosis Factor (TNF) from haemocytes, so TNF exposes phosphatidylserine on tumour cells’ surfaces, causing them to be targeted by cationic defensin, and this interaction leads to cancer cell death and tumour regression [89]. In the same model in which two defensin genes were knocked-out, a significant increase in tumour size was observed [89].

One of the most significant examples of insect AMPs that could have antitumoral potential are those deriving from the insect *Hermetia illucens* L. (Diptera: Stratiomyidae), also known as Black Soldier Fly (BSF), native to the Americas, that represents an extraordinarily rich source of AMPs, due to its ability to live in hostile environments, feeding on decaying substrates rich in microbial colonies [47,48,56,57,59,61].

*H. illucens* AMPs, identified in different transcriptomes, were in silico analysed in order to characterise them for the chemo-physical properties and the putative biological activities, and some of them were predicted as endowed with an anticancer activity (ACPs) [56]. Moreover, the presence of putative ACPs in *H. illucens* was previously predicted in the study of Rajasekhar et al. (2020) [90]: in particular, crude protein extraction from *H. illucens* larvae showed inhibitory activity against MCF7 breast cancer cell line and HeLa cervical carcinoma cell line through cytotoxicity and cell cycle inhibition of G1 and S phase [90]. AMPs are synthesised in different insect tissues and are present in various anatomical structures, mainly in the haemolymph, the insect circulatory fluid where AMPs are released and through which can reach all the organism to combat invading microbes [85,91]. Stimulating larvae with different bacteria can induce specific immune responses, producing peptides capable of significant and various activities, highlighting their potential in the development of new therapies, including in the anticancer field.

A series of research papers investigates various facets of AMPs, with a particular focus on those derived from the BSF, and underscores the potential of these AMPs as alternative therapeutic agents also against antibiotic-resistant bacterial strains [47,61,92,93,94,95]. Also, in works of Park et al., both from 2014 and 2015, the process of immunization of the larvae is essential to first demonstrate that the larval extract possessed a broad spectrum of antibacterial activity, and then the possibility of inducing and purifying novel AMPs using, for example, solid-phase extraction and reverse-phase chromatography [96,97].

The present study evaluates the putative antitumoral properties on GC cell lines of PFs derived from the haemolymph of larvae of *H. illucens*, both uninfected and infected with Gram-positive and Gram-negative bacteria. Our results suggest a mechanism of action similar to that found in the literature for other peptides derived from haemolymph or single peptides of natural or synthetic origin from insects (Coleoptera, Hemiptera, Lepidoptera, Diptera) on HeLa human endocervical cancer cells, U-251 human glioma, VA-13 human lung cancer cells, A375 human melanoma cells, SW1116 human colorectal carcinoma cells, MCF-7 human breast adenocarcinoma cells, HL60 human promyelocytic leukemia cells, and many more [98,99,100,101,102,103,104]., Similar mechanisms have also been observed for peptides from other sources, including terrestrial and marine animals, plant, and synthetic compounds [40,86,105,106,107,108]. In particular, PFs from larvae infected with *E. coli* demonstrated cytotoxicity in both cell lines, with differences in activation or arrest of molecular mechanisms between AGS and KATO III cell lines.

In AGS cells, the activation of apoptotic processes was observed, confirmed by flow cytometric and densitometric analyses, along with associated processes involving cell cycle arrest resulting from the activation of the same apoptotic mechanisms. In contrast, in the KATO III cells, no apoptotic processes were activated; instead, a cell cycle arrest in the S phase was demonstrated, which would explain the cellular response to treatment in the MTT assay. Indeed, a reduction in the ability of cells to metabolize MTT into formazan salts can result from either a decrease in the number of viable cells (cytotoxicity) or a halt in cell proliferation (cytostasis), as may be the case in this instance [109].

The decrease of full-length PARP in a dose-dependent manner was observed in AGS cells treated with PFs. In particular, PARP1, nuclear poly (ADP-ribose) polymerase, appears to be involved in DNA repair in response to environmental stress and is one of the main cleavage targets of Caspase-3, and cleavage of PARP1 facilitates cellular disassembly and serves as a marker of cells undergoing apoptosis. Caspase-3 is a key executioner of apoptosis, being either partially or totally responsible for the proteolytic cleavage of many key proteins, including PARP1. The activation of Caspase-3 requires proteolytic processing of its inactive full-length zymogen (35 kDa) into activated p17 and p12 fragments, therefore the decreasing of full-length Caspase-3 (35 kDa) levels indirectly indicates its fragmentation during the activation of apoptotic processes. Bcl-2 is normally involved in promoting cell survival in response to a wide range of apoptotic stimuli, and a reduction in Bcl-2 levels following treatments may indicate a decreased ability of the cells to resist apoptosis [110,111,112]. Thus, the differential expression of apoptotic markers and variations in cell viability suggest that KATO III cells may be more resistant and possess distinct mechanisms of response to cellular damage compared to AGS cells [113,114]. This was further confirmed by cell cycle analysis, which revealed a differential response between the two cell lines. Indeed, an increase of cells in G2/M, as occurs with AGS cells treated with PF CTR and PF *E. coli*, may indicate a cell cycle arrest prior to mitosis, probably due to DNA damage or other cell cycle arrest signals; in particular, the decrease in cyclin B1 in this case may be explained by its degradation as a result of the inability to progress to mitosis, making it unnecessary to maintain its presence [115,116]. The reduction of p21 as part of a cellular response may indicate a cellular response favouring pro-apoptotic signals when the cell determines that the damage is irreparable and does not proceed to division [117], highlighting its key role in the apoptosis process. Under certain conditions, when the cells are blocked in G2 or G2/M, apoptotic or senescent mechanisms are triggered, as demonstrated by cytofluorimetric and densitometric analyses in the present work, leading to the degradation of p21: this occurs especially in prolonged treatments of cancer cells with DNA damaging agents that can determine a p21 inactivation [118,119]. While an increase of p21 levels in AGS cells treated with PF *M. flavus* could be an indicator of regulation of the DNA damage response, leading to cell cycle arrest and thus preventing the replication of damaged DNA while possibly allowing cells to repair DNA [120], and this could be confirmed also by the cyclin B1 that undergoes a minor reduction, thus indicating that, as cyclin B1 is not directly involved in the S-phase of the cell cycle; therefore, an arrest in this phase would not affect its expression [121]. In contrast, in KATO III cells, the increase of cyclin E2 may be the result of the attempt of the cells to overcome the S-phase dysfunction and promote DNA replication by accumulating cyclin E2 to activate CDK2, a cyclin-dependent CDK2 kinase essential for initiating DNA replication during S-phase [122,123]. In general, compared to untreated cells, this increase in cyclin E2 may be explained by the cells’ attempt to overcome the arrest and maintain or strengthen the signal leading to cell cycle progression and attempt to reactivate the G1 to S transition or complete DNA replication by accumulating cyclin E2. In addition, the increase in p27 levels may be justified as a response to DNA damage or replicative stress induced by PFs, with the aim of blocking cell cycle progression to avoid replication errors [124,125]. This increase can occur before cells stop dividing and begin to differentiate, and may represent a protective mechanism that allows the cell to shut down to trigger repair processes [126].

In summary, our findings show that AGS and KATO III cell lines are sensitive to PFs derived from the larvae infected with *E. coli*, *M. flavus*, and uninfected larvae of the dipteran insect *H. illucens*. The effects of PFs varied depending on their origin, leading to distinct cellular responses, including differences in apoptotic activity and cell cycle arrest at various phases. *E. coli* infection induced the expression of different peptides, compared both to the PF *M. flavus* and PF control, as previously demonstrated [57], so we hypothesize that the major effects detected in PF *E. coli* are due to the peptides differentially expressed. On the contrary, the peptides differentially expressed after *M. flavus* infection, would seem not to have a specific effect on these cell types.

The different effect of PF *E. coli* in some cases may probably be due to several factors, including the varying induction of specific and potent peptides by bacterial infections, whereby Gram-negative bacteria stimulate a strong immune response, activating signalling pathways such as the Imd (immune deficiency) pathway in insects. This pathway is normally activated when specific receptors bind meso-diaminopimelic peptidoglycan-2 (DAP), resulting in a signalling-activation cascade that triggers the transcription of more powerful and specific antimicrobial peptides, which may include compounds that are more cytotoxic to tumour cells [127]. Moreover, it is imperative to emphasise the importance of the characteristics of basal peptides, as peptides produced under normal conditions, i.e., without infection, appear to have a more general activity, acting against a wider range of pathogens, making them more versatile even in an anticancer context [128].

## 4. Materials and Methods

### 4.1. Hermetia Illucens Larvae Infection, Haemolymph Extraction and Peptide Fraction Isolation by Organic Solvents

*Hermetia illucens* (Diptera: Stratiomyidae) larvae were provided by Xflies s.r.l (Potenza, Italy). They were washed with distilled water and 70% ethanol, then infected to increase the production of AMPs. Infection was achieved by injecting the larvae with glass capillaries immersed in a cell suspension of the bacteria *Micrococcus flavus* (Gram-positive, DSM 1790 strain) or of the bacteria *Escherichia coli* (Gram-negative, LGM 2092 strain), both cultured to obtain an optical density (OD) at 600 nm of 1 and incubated at 37 °C and 70% of relative humidity for 24 h. Following incubation, the haemolymph of both infected and uninfected larvae (used as control) was extracted in ice in tubes containing L-ascorbic acid to prevent the melanisation process. The plasma was then recovered, and any debris and cellular components were eliminated by centrifuging the samples for 5′ at 4 °C at 10,000 rpm. The haemolymph protein content was precipitated by organic solvents: methanol (Merck Millipore, Burlington, MA, USA), acetic acid (Merck Millipore, Burlington, MA, USA), and distilled water in a 90:1:9 *v/v* ratio, as reported in Scieuzo et al. [57], in order to obtain a supernatant containing peptides with a molecular weight lower than 30 kDa. The supernatant was re-suspended in a volume of sterile water equal to that of the initially collected haemolymph and allowed to air-dry to remove organic solvents. The resulting samples, thus prepared, correspond to peptide fractions (PFs) derived from haemolymph extracted from uninfected larvae (PF CTR) and from larvae infected with *E. coli* (PF *E. coli*) and *M. flavus* (PF *M. flavus*). To remove possible traces of lipids that could be co-extracted due to the use of methanol, a further treatment with hexane was performed. An equal volume of hexane (Merck Millipore, Burlington, MA, USA) was added to each extract, and the samples were then vortexed and centrifuged at 16,000 rcf for 20 min at 4 °C [57]. The upper fraction, possibly containing lipids, was removed. The Bradford assay was used to determine the quantity of peptides in each sample and establish experimental concentrations [129].

### 4.2. Cell Lines and Culture Conditions

KATO III (RRID:CVCL_0371) and AGS (RRID:CVCL_0139) human gastric carcinoma and adenocarcinoma cell lines, respectively, were used for in vitro studies and were purchased from American Type Culture Collection (ATCC, Manassas, VA, USA).

KATO III were cultured in Iscove’s Modified Dulbecco’s Medium (IMDM, Gibco™, Grand Island, NY, USA) with *L*-glutamine and supplemented with 20% Fetal Bovine Serum (FBS) and 1% penicillin-streptomycin (Euroclone, Milan, Italy), and AGS were cultured in Dulbecco’s Modified Eagle’s Medium (DMEM)—low glucose (1 g/L) (IMDM, Gibco™, Grand Island, NY, USA) with *L*-glutamine and 110 mg/L Sodium Pyruvate—and supplemented with 10% Fetal Bovine Serum (FBS) and 1% penicillin-streptomycin, respectively, in a humid 5% CO_2_ atmosphere at 37 °C. Every two to three days, cells were split, while the medium was refreshed almost every day.

### 4.3. Measurement of Cell Viability

Cell viability was evaluated by using the MTT [3-(4,5-dimethylthiazol2-yl)-2,5-diphenyltetrazolium bromide] (Sigma-Aldrich, Inc., Saint Louis, MO, USA) colorimetric assay. AGS and KATO III cells were seeded in 96-well microtiter plates (5 × 10^3^ cells per well) and incubated for 24 h. Medium was then replaced to treat cells for 24, 48, and 72 h, and to prepare decreased concentrations (20 µg/mL, 10 µg/mL, 5 µg/mL, 2.5 µg/mL, 1.25 µg/mL, 0.62 µg/mL, 0.31 µg/mL, 0.16 µg/mL) of PF (1 µg/µL), diluted into the specific culture medium for each cell type, obtained from larvae infected with *E. coli* (PF *E. coli*), with *M. flavus* (PF *M. flavus*), and from uninfected larvae (PF CTR). The concentrations of 20 µg/mL, 10 µg/mL, and 2.5 µg/mL were used to assess all the assays, all at 48 h of treatments. Untreated cells were considered as control. At the end of the treatment, the medium was aspirated and the plate incubated for 4 h in the dark at 37 °C and 5% CO_2_ after adding to each well 100 µL of the 0.75 mg/mL MTT (Sigma Aldrich-Merck^®^) solution, starting from a stock solution of 5 mg/mL. After 4 h, this solution was aspirated, noting the formation of formazan crystals remaining at the bottom, immediately solubilised in 100 µL of the stop solution (composed by isopropanol and DMSO in a 1:1 ratio + 1% Triton) and the plates were incubated at 37 °C for 1–2 h until the complete dissolution of the salts. The amount of MTT formazan crystals was evaluated by measuring absorbance using a VICTOR^®^ NivoTM multimode plate reader (PerkinElmer^®^, Shelton, CT, USA) at a wavelength of 570 nm. The 50% inhibitory concentration (IC50) was then calculated (Supplementary File S1).

From this point onwards, the cells were treated for 48 h with concentrations of 20 µg/mL, 10 µg/mL, and 2.5 µg/mL of each sample in all subsequent experiments. The 48 h treatment time was selected because it would allow significant effects of PFs on the cells to be observed, while maintaining a time window in which the cells themselves respond to the treatment without the effects becoming too extreme as seen in prolonged treatments. The concentrations were chosen to be close to the IC50, with the aim of including a concentration capable of producing a significant effect (20 µg/mL), which would allow a clear response to be observed in the cells, a concentration in which the cells would give a response as close as possible to the untreated control (2.5 µg/mL), and a concentration intermediate between the two previous mentioned (10 µg/mL).

### 4.4. Observation of Morphological Changes

AGS (2.5 × 10^5^ cells per well) and KATO III (4 × 10^5^ cells per well) cells were seeded into 6-well plates and cultured overnight. Then, they were treated for 48 h with different concentrations of PFs (20 µg/mL, 10 µg/mL, 2.5 µg/mL), using untreated cells as control. The cellular morphology was observed using the inverted microscope Axio Vert.A1 (Zeiss™, White Plains, NY, USA).

### 4.5. Cytofluorimetric Analysis for Apoptosis Assay

AGS cells (2.5 × 10^5^ cells per well) and KATO III cells (4 × 10^5^ cells per well) were seeded in 6-well plates, incubated for 24 h and treated with PFs (20 µg/mL, 10 µg/mL, 2.5 µg/mL). Untreated cells were considered as control. After 48 h of treatment, cells were washed once with cold 1X PBS, harvested and collected. Each sample was resuspended in 100 µL of 1X Annexin Binding Buffer at a concentration of 1 × 10^6^ cells/mL with 5 µL of FITC Annexin V and 5 µL of Propidium Iodide (PI) Staining Solution of the Apoptosis Detection kit I (BD Pharmigen™, San Jose, CA, USA), according to the manufacturer’s instructions, and incubated in the dark for 15 min at room temperature. Then, 400 µL of 1X Annexin Binding Buffer was added to each tube and the analysis was carried out by DxFLEX Flow Cytometer (Beckman Coulter, USA). Data were analysed with the Kaluza software V1.1.2 (Beckman Coulter Diagnostics, Brea, CA, USA). For every sample, 10,000 events in total were acquired. The mean ± SE (Standard Error) was used to report the data from treated samples, which were normalised using the fold change of untreated controls of a minimum of three separate experiments.

### 4.6. Cytofluorimetric Analysis for Cell Cycle Assay

AGS and KATO III cells were seeded in 6-well plates at a confluence of 2.5 × 10^5^ and 4 × 10^5^ cells per well, respectively, and incubated for 24 h at 37 °C at 5% CO_2_. After 48 h of treatment with PFs (20 µg/mL, 10 µg/mL, 2.5 µg/mL), cells were harvested, washed with PBS, and fixed in ice-cold 70% ethanol for 1 h. Fixed cells were then incubated with PI/RNaseA staining solution for 30 min at room temperature and in the dark. The acquisition was performed by DxFLEX Flow Cytometer (Beckman Coulter, USA) and the analysis was conducted using the Kaluza analysis software 2.1.

### 4.7. Western Blot Analysis

AGS cells (2.5 × 10^5^ cells/well) and KATO III cells (4 × 10^5^ cells/well) were seeded into 6 well plates and cultured overnight. They were treated for 48 h with PFs (20 µg/mL, 10 µg/mL, 2.5 µg/mL). Harvested cells were lysed using Pierce™ RIPA Lysis (Pierce Biotechnology Inc., Rockford, IL, USA) and Extraction Buffer (Thermo Scientific™, Segrate (MI), Italy) supplemented with Halt™ Protease Inhibitor Cocktail 1X (Thermo Scientific™). Protein concentration was quantified using the Bradford Reagent (Bio-Rad, Hercules, CA, USA), with Bovine Serum Albumin (BSA) as the standard. Equal amounts of cell lysates (20 µg) were resolved by SDS-PAGE (Sodium Dodecyl Sulfate PolyAcrylamide Gel Electrophoresis) through 4–20% poly-acrylamide gels. After transferring the proteins to PVDF blotting membranes using a Trans-Blot^®^ Turbo™ Transfer System (Bio-Rad, Hercules, CA, USA), the membranes were blocked with 5% non-fat milk (Skim Milk Powder, Sigma-Aldrich, Saint Louis, MO, USA) in 1X PBST (Phosphate buffered saline (PBS) washing buffer for peroxidase conjugates in Western Blotting with 0.1% TWEEN^®^ 20, Sigma-Aldrich) for 1 h. Then, the membranes were probed overnight at 4 °C with the following primary antibodies: PARP (46D11) Rabbit mAb (#9532; dilution 1:1000; Cell Signaling Technology^®^, Danvers, MA, USA); Caspase-3 Antibody (#9662; dilution 1:1000; Cell Signaling Technology^®^); Bcl-2 (50E3) Rabbit mAb (#2870; dilution 1:1000; Cell Signaling Technology^®^); Cyclin E2 Antibody (#4132; dilution 1:1000; Cell Signaling Technology^®^); p21 Waf1/Cip1 (12D1) Rabbit mAb (#2947; dilution 1:1000; Cell Signaling Technology^®^); p27 Kip1 (D69C12) Rabbit mAb (#3686; dilution 1:1000; Cell Signaling Technology^®^); cyclin B1 (G-11) (sc-166757; dilution 1:1000; Santa Cruz Biotechnology, Inc., Dallas, TX, USA); β-Actin (8H10D10) Mouse mAb (#3700; dilution 1:1000; Cell Signaling Technology^®^), and Vinculin (7F9) (sc-73614; dilution 1:1000; Santa Cruz Biotechnology, Inc.). Then, the membranes were washed three times using wash buffer (1X PBST) and incubated for 1 h with the corresponding HRP-conjugated secondary antibody: Anti-mouse IgG-HRP-linked (#7076; dilution 1:3000; Cell Signaling Technology^®^), and Anti-rabbit IgG-HRP-linked (#7074; dilution 1:3000; Cell Signaling Technology^®^). Finally, they were visualised using the enhanced chemiluminescence kit for Western blotting detection Clarity™ Western ECL Substrate from Bio-Rad Laboratories and acquired with the ChemiDoc detection system (Bio-Rad, Hercules, CA, USA) using ImageLab software 1.53t. Densitometric analysis of the bands was performed using ImageJ software 1.53t (http://imagej.nih.gov/ij, accessed on 22 December 2024).

### 4.8. Statistical Analysis

Data are presented as means ± Standard Error (SE) of three technical replicates of three independent assays. Data were set to fit a normal distribution. Statistical significance between groups was evaluated by one-way analysis of variance (ANOVA), followed by Dunnett’s *post hoc* test, performed using GraphPad Prism version 5.00 for Windows (www.graphpad.com). The difference was considered statistically significant at * *p* < 0.05, ** *p* < 0.01, *** *p* < 0.001, ^#^ *p* < 0.05, ^##^ *p* < 0.01, ^###^ *p* < 0.001, ^$^ *p* < 0.05, ^$$^ *p* < 0.01, ^$$$^ *p* < 0.001.

## Data Availability

The data sets used and/or analysed during the current study are available from the corresponding author on reasonable request.

## Supplementary Materials

The supporting information can be downloaded at: https://www.mdpi.com/article/10.3390/ijms26051885/s1.
